# Potential Impact of Combined Inhibition by Bacteriocins and Chemical Substances of Foodborne Pathogenic and Spoilage Bacteria: A Review

**DOI:** 10.3390/foods12163128

**Published:** 2023-08-20

**Authors:** Wei Yu, Jinqi Guo, Yuanyuan Liu, Xiaoge Xue, Xiangru Wang, Lili Wei, Jiage Ma

**Affiliations:** Key Laboratory of Dairy Science, Ministry of Education, College of Food Science, Northeast Agricultural University, 600 Changjiang Road, Harbin 150030, China; yuwei_www@163.com (W.Y.); guojinqi2022@163.com (J.G.); 15660856409@163.com (Y.L.); xiaoage20212022@163.com (X.X.); xiangru23@163.com (X.W.); 13028670259@163.com (L.W.)

**Keywords:** nisin, chemicals, combination, antibacterial mechanism

## Abstract

In recent years, food safety caused by foodborne pathogens and spoilage bacteria has become a major public health problem worldwide. Bacteriocins are a kind of antibacterial peptide synthesized by microbial ribosomes, and are widely used as food preservatives. However, when used individually bacteriocins may have limitations such as high cost of isolation and purification, narrow inhibitory spectrum, easy degradation by enzymes, and vulnerability to complex food environments. Numerous studies have demonstrated that co-treatment with bacteriocins and a variety of chemical substances can have synergistic antibacterial effects on spoilage microorganisms and foodborne pathogens, effectively prolonging the shelf life of food and ensuring food safety. Therefore, this paper systematically summarizes the synergistic bacteriostatic strategies of bacteriocins in combination with chemical substances such as essential oils, plant extracts, and organic acids. The impacts of bacteriocins when used individually and in combination with other chemical substances on different food substrates are clarified, and bacteriocin–chemical substance compositions that enhance antibacterial effectiveness and reduce the potential negative effects of chemical preservatives are highlighted and discussed. Combined treatments involving bacteriocins and different kinds of chemical substances are expected to be a promising new antibacterial method and to become widely used in both the food industry and biological medicine.

## 1. Introduction

The rapid growth of the global population has brought great challenges to the food industry [1,2]. It is noteworthy that microbial contamination caused by foodborne pathogens and spoilage microorganisms is one of the greatest potential risks to food safety [3]. Foodborne bacteria and spoilage microorganisms cause great economic losses in the process of food processing, transportation, and storage, and are considered a main public health problem in both developing and developed countries [4,5]. Foodborne diseases are caused by ingestion of food contaminated by bacteria, fungi, viruses, and other microorganisms, as well as by physical and chemical factors [6]. Methicillin-resistant *Staphylococcus aureus* (*S. aureus*), pathogenic *Escherichia coli* (*E. coli*), *Salmonella typhimurium* (*S. typhimurium*), and *Listeria monocytogenes* (*L. monocytogenes*) have attracted great attention, as they may lead to many diseases, including colitis, intestinal dysfunction, and pneumonia [7,8,9]. According to a report of the World Health Organization, in 2010 about 600 million people in the world became sick and 420,000 died from eating contaminated food [10]. Benzoic acid, sorbic acid, nitrite, and parabens are usually used to inhibit the growth of pathogens and spoilage microorganisms in food [11,12]. Nevertheless, it has been reported that the hormone system and the physiological function of organisms may be interfered with by parabens [13]. Furthermore, nitrite can combine with hemoglobin to form methemoglobin, which leads to blue baby syndrome and excessive intake of nitrite has potential carcinogenic risk [14]. Although preservatives have been extensively tested on animals before being approved for use, there are differences between the intestines of experimental animals and those of humans. Notably, dietary factors greatly affect the flora structure and function of intestinal microorganisms [15,16]. In addition, studies have found that chemical preservatives are related to obesity, allergies, asthma, etc. [17]. For instance, although potassium sorbate and sodium sorbate are internationally recognized safe preservatives, excessive intake negatively affects the body’s metabolic balance [18]. Furthermore, food processing technologies may destroy the structure of the original nutrients of the food, and high acidity during food processing can affect the structure of DNA [19]. Therefore, long-term consumption of chemical preservatives may be potentially harmful to human health. In addition to chemical preservatives, antibiotics can be used [20]. However, more and more bacteria have developed resistance to the existing antibiotics, and the spread and prevalence of antibiotic resistance genes has become a major problem in the 21st century [21,22]. Data from the 2020 Global Antibiotic Resistance and Use Surveillance System declared that high rates of problematic antibiotic resistance occurred in 66 countries [23]. The risks caused by antibiotics and antibiotic resistance cannot be underestimated [24]. 

Therefore, researchers are committed to exploring green, safe, and effective natural substances as substitutes for chemical preservatives and antibiotics [25]. With people paying more and more attention to food safety and quality, consumers are demanding that fresh food should have the least processing and most natural preservatives [26,27]. In past decades, bacteriocin, has been widely used as a food biopreservative in the food industry because of its safety and easy digestion in the gastrointestinal tract [28,29,30]. Bacteriocin is an antibacterial peptide synthesized by ribosomes which has inhibitory activity against gram-positive bacteria and gram-negative bacteria [31]. Bacteriocins may penetrate cell membranes and cause leakage of cell contents such as K^+^ and inorganic phosphate, or exert a bacteriostatic effect by blocking the biosynthesis of DNA, RNA, and proteins [32,33]. Previous studies have shown that the cell membrane of gram-negative bacteria is composed of an outer membrane, cell wall, peptidoglycan, and inner membrane [34]. Due to the protective barrier of the outer membrane, bacteriocin cannot reach its target, peptidoglycan precursor lipid II [35]. Therefore, gram-negative bacteria are usually insensitive to bacteriocin. Currently, bacteriocins used as food preservatives include nisin, natamycin, subtilin, pediocin, tylosin, and carnocyclin A [36,37,38]. In addition, researchers have used different types of bacteriocins in combination and obtained a better synergistic antibacterial effect. Nisin A combined with lacticin 3147 can synergistically inhibit the growth of *Listeria innocua* in cheese [39]. The combination of enterocin LD3 and plantaricin LD4 can synergistically destroy the cell membranes of *S. aureus* ATCC25923 and *S. typhimurium* ATCC13311 [40]. Among these bacteriocins, nisin is widely used and has been approved as a food preservative by more than 50 countries [41,42]. The safety of nisin has been approved by the United States Food and Drug Administration (FDA) [43]. Nisin is considered as a Generally Recognized as Safe (GRAS) ingredient [44]. It is active against a wide range of gram-positive bacteria and spores, and does not produce cross-resistance to bacteria. It is speculated that different kinds of bacteriocins may not produce cross-resistance to bacteria because they have different structures and action targets [45]. As a natural and safe antibacterial agent, nisin has been widely used in dairy products, meat products, fruits, and vegetables [46,47]. However, its low solubility, uneven distribution, and interaction with food ingredients leads to the decline of its antibacterial activity [48,49]. Therefore, there exists an urgent need to improve the stability of bacteriocin in the food system in order for it to play a better role in bacteriostasis. 

It has been found that chemical substances such as essential oils (EOs), plant extracts, and organic acids (OAs) have the ability to inhibit the growth of foodborne pathogens and spoilage microorganisms. Pingyin rose bud extract has been used as a natural antibacterial agent against *S. aureus* ATCC 25923 [50]. Phenyllactic acid reduced the metabolic activity and viability of cells, destroyed the integrity of cell membrane, and caused the leakage of intracellular contents such as DNA, protein, and ATP of Staphylococcus aureus CICC 10145 [51]. Therefore, the combination of bacteriocin and other chemical substances is an effective strategy to control the growth of foodborne pathogens in complex food environments, reduce the drug resistance of bacteriocin, expand the antibacterial spectrum, and improve the antibacterial effect [52]. In this paper, we systematically elucidate the bacteriostatic effects of bacteriocin combined with other chemical substances. In addition, we preliminarily summarize their mechanism of action and effects on food. It is necessary to strengthen the existing research on bacteriocin and its combined action with various chemical substances in order to promote the application of bacteriocin in the food industry. 

## 2. Concept, Classification, and Mode of Action of Bacteriocins

### 2.1. Concept of Bacteriocins 

Bacteriocin is an antibacterial polypeptide or protein synthesized by bacteria through ribosomes during metabolism [53]. Most bacteriocins have the ability to inhibit gram-positive bacteria, fungi, and viruses [54]. Studies have shown that nisin affects the production of reactive oxygen species in cells of *Bacillus subtilis* (*B. subtilis*) and *S. aureus* through the mitochondrial electron transport chain [55]. A novel bacteriocin CAMT6 produced by *Enterococcus durans* YQ-6 isolated significantly removed biofilms produced by *L. monocytogenes* ATCC 19111 and inhibited *L. monocytogenes* by disrupting the permeability of the cell membrane (*p* < 0.05) [41]. In addition to their antimicrobial effects, certain bacteriocins show other biological activities, such as anti-inflammation, anti-oxidation, regulating intestinal flora, and improving colitis [56,57,58]. Studies have shown that most bacteriocins possess good acid and alkali resistance, high thermal stability and efficiency, nontoxicity, no residue, and no induced drug resistance [59,60]. Among the discovered bacteriocins, bacteriocins from lactic acid bacteria (LAB) are superior in terms of safety to bacteriocins produced by *Enterococcus*, *Pediococcus*, and *Leuconostoc* strains [61,62]. Most LABs are recognized to be GRAS by the FDA [63]. LAB bacteriocins have suitable characteristics to be used as food preservatives [64].

### 2.2. Classification of Bacteriocins

The classification parameters of bacteriocin mainly include molecular weight, mode of action, antibacterial activity, enzymatic stability, chemical structure, and the existence of modified amino acid residues after translation [65]. Bacteriocins can be divided into four categories according to their molecular weight, structure, mode of action, and function (Table 1) [54]. Class I bacteriocins are post-translational modified peptides that contain unusual amino acids such as lantibiotics and derivatives, and their molecular weight is usually lower than 5 kDa [66,67]. Nisin is produced by standard *Lactococcus lactis* (*L. lactis*), which is a typical example of a Class I bacteriocin, and was confirmed as GRAS by the FDA in 1988 [68]. Nisin is a widely studied and applied bacteriocin in the food industry, and is characterized by a wide spectrum of antibacterial activity against gram-positive bacteria [69]. It is widely used as a food additive in meat, dairy products, vegetables, and canned foods [70]. Class II bacteriocins comprise a very large group of heat-stable unmodified peptide bacteriocins, which can be further divided into four subgroups [71]. The class IIa bacteriocins are known as pediocin-like bacteriocins; this is currently the largest and most extensively studied subgroup of class II bacteriocins [72]. The class IIb bacteriocins, called dipeptide class II bacteriocins, are composed of two different individual peptides; about the same amount of each peptide is required to exert its best antimicrobial activity [73]. The class IIc bacteriocins, known as circular bacteriocins, are characterized by a peptide bond that links the N- and C-termini of the core peptide [74]. The class IId bacteriocins are single linear peptides [75]. Class III bacteriocins include heat-labile proteins, and their molecular weight is more than 10 kDa [76]. Helveticin J, Helveticin M, colicin, and enterolysin A are typical Class III bacteriocins, and were produced by *Lactobacillus helveticin*, *Lactobacillus crispatus*, *E. coli*, and *Enterobacter faecalis*, respectively [77]. It has been found that helveticus M can destroy the cell wall of gram-positive bacteria and the outer membrane of gram-negative bacteria, making it effective against both types [78]. Class IV bacteriocins are complex macromolecular compounds that include proteins, carbohydrates, or lipids. At present, these bacteriocins have not been purified [79]. 

### 2.3. Mode of Action of Bacteriocins

**Figure 1:**
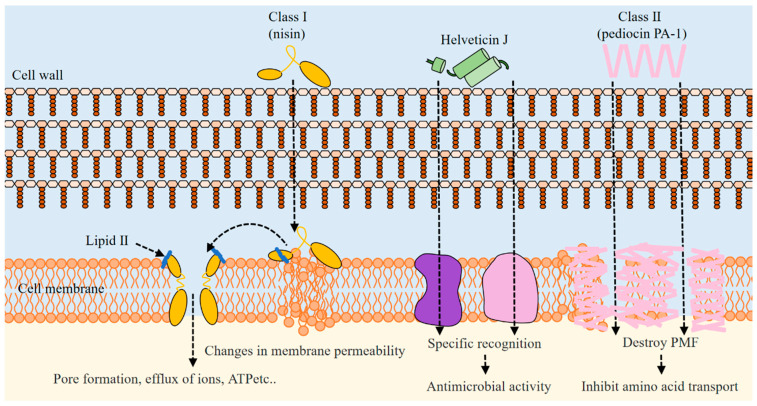
Schematic diagram of bacteriocin mechanism of action.

The mechanism of action of bacteriocin depends on the structure of the specific peptide, the producing strain, and the dose of bacteriocin [86]. In one of the most widely recognized mechanisms, bacteriocin binds to specific receptors on the cell membrane, which makes the cell membrane form pores, leading to the leakage of cell components of the target bacteria and dissipation of the proton motive force (PMF) [87]. Different types of bacteriocins have different mechanisms of action on biofilms [88]. Class I cationic lantibiotics bind to the target cells by electrostatic force, then interact with the precursor substances of lipid II on the target cell wall and peptidoglycan to form a complex of bacteriocin and lipid II. Subsequently, the complex of bacteriocin and lipid II attaches to the cell membrane of the target bacteria, forming pores in the cell membrane and promoting the outflow of Mg^2+^ and Ca^2+^ ions [89]. Class II bacteriocins recognize the mannose phosphate transferase system through the N-terminal β-fold region, then the protein complex accelerates the flow of carbohydrates on the surface of the cell membrane, resulting in the formation of hydrophilic pores in the cell wall, promoting α-helix amphiphilic C-terminal domain channels, and ultimately disrupting PMF and inhibiting amino acid transport, which induces cell death [90]. Class I bacteriocins form pores in a “wedge-like” model, while class II bacteriocins increase membrane permeability through “barrel stave” pores or a “carpet” mechanism [91]. The mechanism of action of bacteriocins is illustrated in Figure 1.

## 3. Combinations of Bacteriocins and Chemical Substances

**Figure 2:**
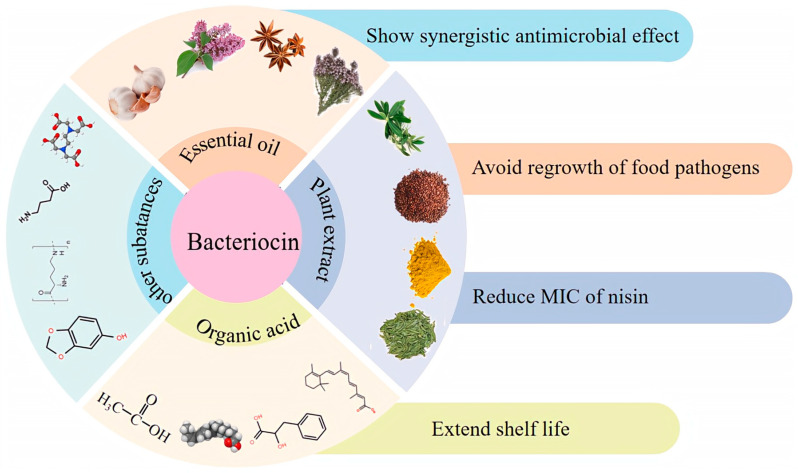
Combined effects of bacteriocins and chemical substances.

Due to enzyme degradation, poor solubility, uneven distribution in food, and partial inactivation through interaction with food components, the efficiency of bacteriocins is weakened when directly added to food systems [92]. Therefore, it is necessary to expand the antibacterial spectrum of bacteriocins, enhance their antibacterial effect, and avoid drug resistance. The synergistic antibacterial effect of bacteriocin and chemical substances has aroused widespread interest. Numerous studies have demonstrated a stronger antibacterial effect when bacteriocins are combined with chemical substances such as EOs, plant extracts, and OAs compared to when used alone (Figure 2). 

### 3.1. Bacteriocins and Essential Oils (EOs) 

EOs are a kind of plant secondary metabolite with a characteristic aroma; they are separated from aromatic plants as volatile oily liquids [93]. EOs gained from plants and spices are rich in phenols, flavonoids, anthocyanins, and terpenoids, which possess antibacterial and antioxidant effects [94]. The methods used to extract EOs mainly include distillation, pressing, water diffusion, and organic solvent extraction [95]. EOs are classified as GRAS compounds by the United States FDA, and have been approved as food additives or spices [96]. At present, studies have shown that EO can inhibit the growth of pathogenic bacteria such as *S. aureus* and *L. monocytogenes* [97]. EO showed antibacterial activity by destroying biofilm barriers, inhibiting the secretion of extracellular polymeric substances and protein, changing ATP concentration and cytoplasmic pH, and controlling genes in an Agr system [98]. Oregano and cinnamon EOs used as natural alternatives inhibited the proliferation of *L. monocytogenes* ATCC 7644 [99]. It has been reported that *Thuja koraiensis Nakai* EO and citrus EO have antibacterial activity against *S. aureus*, *E. coli*, and *B. subtilis* [100,101]. The biofilm formation of different strains of *L. monocytogenes* in chicken juice, a lettuce leaf model medium, and skim ultra-high-temperature milk was inhibited by *Thymus zygis* EOs during 14 days of storage [102]. 

To control the incidence of foodborne illnesses and the use of synthetic additives, different combinations of bacteriocins and EOs have been widely studied. As shown in Table 2, combining bacteriocins and EOs can reduce the risk of spoilage and presence of pathogenic bacteria in various foods, as well as prolong their shelf life. Wang et al. found that nisin and *Perilla frutescense* EO invaded the cell membrane of *S. aureus*, *E. coli*, *Salmonella enteritidis* (*S. enteritidis*), and *Pseudomonas tolaasii* (*P. tolaasii*) to form cavities, resulting in cytoplasm outflow, cell lysis, and death [103]. The fractional inhibitory concentration index (FICI) confirmed that nisin combined with carvacrol had a synergistic effect on *L. monocytogenes* 10403S [104]. Another study showed that the antibacterial activity of the combined action of nisin and carvacrol was better against standard *S. aureus* BNCC 186,335 in pasteurized milk than that of nisin and carvacrol alone at 25 °C and 4 °C. Moreover, this combined formula could lead to membrane damage of *S. aureus* and leakage of nucleic acid and protein [105]. The combination of nisin and garlic EO reduced the number of viable bacteria of *L. monocytogenes* ATCC 19118, which was possibility due to the addition of EO increasing the number and size of pores on the cell membrane compared with nisin alone [106]. T. Mehdizadeh et al. found that nisin combined with *Ocimum basilicum*, *Salvia officinalis*, and *Trachyspermum ammi* EOs inhibited the growth of *E. coli* O157 by altering the permeability of the membrane, the proton motive force, the efflux of amino acid, and the pH gradient of bacteria [107]. Enteriocin A has been used as a food biopreservation because of its potent antimicrobial activity against *L. monocytogenes* [108]. *L. monocytogenes* could not be detected after the combined action of enterocin A and *Thymus vulgaris* EOs at 37 °C for 18 h [109]. In addition, studies have focused on the fabrication of packaging films loaded with bacteriocin and EOs to reduce spoilage bacteria and pathogenic microorganisms in various food [110]. Chitosan-based edible films incorporated with nisin and clove EO inhibited the growth of psychrotrophic, Enterobacteriaceae, and LAB. Compared with chitosan-based edible films incorporated with nisin and EO only, the total viable count (TVC) when incorporated with nisin and EO increased more slowly [111].

The combination of bacteriocin and EO is expected to improve the sensory qualities of food during storage and extend shelf life [112]. Nisin combined with oregano EO significantly extended the shelf life of fish fillets from 16 days to 28 days when compared with nisin or EO used individually [113]. A nano-emulsion-based active coating (NEAC) doped with nisin, star anise EO, and polylysine inhibited the growth of *E. coli* and TVC. NEAC extended the shelf life of Yao meat from 8 days to 16 days. Moreover, Yao meat with NEAC showed the best overall acceptance [114]. 

Thus, bacteriocin and EO may inhibit the growth of pathogens and spoilage bacterium by changing membrane permeability and PMF or destroying the cell membrane, while prolonging the shelf life of food without adversely affecting its overall sensory acceptance. The synergistic effect of bacteriocins combined with essential oils may be attributed to the fact that bacteriocin and essential oil have different targets. Thus, the combination of bacteriocin and EO can act on two or more targets at the same time, exerting a stronger antibacterial effect [115]. EOs have the functions of regulating immunity, anti-inflammation, and anti-oxidation [116]. Therefore, EOs can be used to supplement bacteriocin, which can enrich the growth of anti-oxidative stress bacteria, reduce oxidative stress, and improve the inhibition of colitis [58]. At the same time, their combination can meet consumer demands for more natural and preservative-free foods. The combination of bacteriocin and EO may be of assistance to the development of antimicrobials for the food industry.

**Table 2:** Application of bacteriocins in combined action with essential oils (EO).

Bacteriocins	EO	Food System	Target Microorganisms	Result	References
Nisin	*Perilla frutescense* EO	Strawberry	*S. aureus*; *E. coli*; *S. enteritidis*; *P. tolaasii*	Cytoplasmic efflux, cell lysis and death	[103]
Nisin	Carvacrol	Sliced bologna sausage	*L. monocytogenes* 10403S	Retards bacterial reproduction	[104]
Nisin	Carvacrol	Pasteurized milk	*S. aureus* BNCC 186,335	Cell membrane damage, nucleic acid, protein leakage	[105]
Nisin	Garlic EO	—	*L. monocytogenes* ATCC 19118	The cell membrane forms pores	[106]
Nisin	*Ocimum basilicum*, *Salvia officinalis* and *Trachyspermum ammi EOs*	—	*E. coli* O157 (ATCC 25922)	The membrane permeability, proton motility, efflux of amino acids and pH gradient of bacteria are changed	[107]
Enteriocin A	*Thymus vulgaris* EOs	—	*L. monocytogenes*; *E. coli* O157:H7	Inhibition of bacterial growth	[109]
Nisin	Clove EO	Pork patties in cold storage	TVC; Psychrotrophs; Enterobacteriaceae; LAB	Inhibit the growth of psychrophilic bacteria, Enterobacteriaceae and LAB in pork samples	[111]
Nisin	Oregano EO	Grass carp (*Ctenopharyngodon idellus*)	TVC	The storage period was prolonged, and the TVC decreased	[113]
Nisin	Star anise EO	Ready-to-eat Yao meat products	*E. coli*	Increase the pH value of pork and volatile salt base total nitrogen, delay the proliferation of *E. coli*, prolong the shelf life	[114]

### 3.2. Bacteriocins and Plant Extracts 

Plant extracts are substances extracted or processed from all or part of plants, and can be used in health food, dietary supplements, medicine, and other industries [117,118]. At present, the main extraction methods are reflux extraction, ultrasonic extraction, microwave extraction, and supercritical fluid extraction [119]. Plant extracts exhibit strong biological activities, such as immune regulation, anti-infection, anticoagulation, antioxidant capacity, anti-inflammatory, and can improve gastrointestinal health [120]. A study reported that the active ingredients of plant extracts have antibacterial effects such as polyphenols, flavonoids, polysaccharides, and terpenoids. However, due to the different sources of plant extracts and tested bacteria, it is difficult to elucidate their mechanisms from a single aspect [121]. At present, the bacteriostatic mechanisms of plant extracts mainly include the following aspects: (i) changing the permeability of the cell membrane; (ii) destroying the enzyme system in bacterial cells; and (iii) affecting the metabolic pathway of bacterial oxidation and respiration [122,123]. Furthermore, it has been found that plant extracts can inhibit microbial growth and extend the shelf life of food. Olive leaf extract inhibited the growth of staphylococcal, mold, yeast, psychrophilic bacteria, and total aerobic bacteria in poultry meat by disrupting the cytoplasmic membrane, damaging membrane proteins, and interfering with membrane-integrated enzymes [124]. Using a chitosan/guar gum film matrix and active packaging film of walnut green husk extract (*Juglans regia* L.) inhibited the growth of yeast and mold and reduced the occurrence of shrinkage and browning of fresh-cut apples caused by rapid cell dehydration and cell wall hydrolysis [125]. 

In recent years, many studies have proven that bacteriocin combined with plant extracts can inhibit the growth and reproduction of pathogenic bacteria, prolong the shelf life of food, and provide nutrients such as polyphenols, polysaccharides, terpenoids, flavonoids, and alkaloids to consumers (Table 3) [126,127]. Studies have shown that the combination of grape seed extract (GSE) and bacteriocin has a synergistic antibacterial effect. Nisin combined with GSE reduced the number of standard *L. monocytogenes* SSA184 by 2.96 log CFU/g by blocking the TCA cycle, amino acid biosynthesis, and energy metabolism at 37 °C for 24 h [128]. The research team inoculated nisin (2000 IU/mL) and GSE (1%, *w*/*v*) on cooked shrimp (*Litopenaeus vannamei*). Notable reductions of standard *L. monocytogenes* (SSA184, SSA97, and LM10) were observed after 15 min treatment, along with a reduction range of 1.7~1.9 log CFU/g [129]. In conclusion, the combination of nisin and GSE could be a promising strategy for the food industry to control contamination of *L. monocytogenes* and maintain food safety. Conversely, studies have found that the combined use of plant extracts and nisin does not inhibit microbial reproduction in meat. A chitosan–gelatine edible coating loaded with nisin and GSE did not improve the antimicrobial effect, and the TVC values of all coated and non-coated fresh pork increased significantly at 4 °C during 20 days. This may be caused by the growth of psychrotrophic bacteria such as *Carnobacterium* spp. and *Pseudomonas* spp. [130]. This controversial result reveals that nisin and GSE have different effects on different food matrices, inhibition mechanisms, temperature, environment, and pH; thus, it is important to comprehensively consider and choose appropriate chemical substances to combine with nisin. 

Phosphatidylcholine nanoliposomes co-encapsulated with nisin and garlic extract (*Allium sativum* L.) reduced *L. monocytogenes* ATCC 7644 by about 40.86% in milk [131]. Nisin combined with thymoquinone had a better combined bactericidal effect on *L. monocytogenes* ATCC 19115 and ATCC 15313 in Tryptone Soy Broth sterilized milk than nisin and thymoquinone alone [132]. It was found that nisin combined with green tea extract could significantly inhibit the growth of *E. coli* ATCC 25922, mesophilic bacteria, and yeast (*p* < 0.05) [133]. In addition, the combination of plant extracts and nisin can improve the texture and sensory characteristics of food. The rapid increase of thiobarbituric acid-reactive substances and metmyoglobin in camel meat was inhibited using nisin and *Olea europaea* subsp. *laperrinei* leaf extract [134]. Meral et al. successfully prepared nisin and curcumin loaded nanomaterials (NCL) with an average diameter of 172 nm. It was found that the total thermophilic aerobic bacteria and LAB of fish fillets coated with NCL nanoparticles were lower than those of the uncoated control, and that the hardness of fish fillets coated with nisin and curcumin nanomaterials was higher than that of the control during 12 days at 4 °C [48]. 

In summation, the combination of bacteriocin and plant extracts can inhibit the proliferation of foodborne pathogens and spoilage bacteria by changing cell membrane permeability, leading to protein or nucleic acid leakage and inhibiting metabolic pathways. Selected plant extracts destroy the bacterial cell membrane, which leads to increased permeability. At the same time, bacteriocin enters bacterial cells or passes through biofilms using short-term changes in membrane permeability, then accumulates in the bacterial plasma membrane using its hydrophobicity. This causes various toxic effects, hindering bacterial metabolism and interfering with various metabolic pathways [135]. Therefore, use of bacteriocins in combination with plant extracts may be a promising method of maintaining the quality of food products and prolonging their shelf life. In addition, due to differences in the environment or other action conditions, the combined effects of the same plant extract and bacteriocin may be the opposite; this difference deserves further study. 

**Table 3:** Application of bacteriocins in combined action with plant extracts.

Bacteriocins	Plant Extract	Food System	Target Microorganisms	Result	References
Nisin	Grape seed extract	Shrimps	*L. monocytogenes* (stereotype 3a, SSA184)	Protein and nucleic acid leakage	[128]
Nisin	Grape seed extract	Shrimps	*L. monocytogenes* (SSA184, SSA97 and LM10)	The number of *L. monocytogenes* decreased	[129]
Nisin	Grape seed extract	Fresh pork	TVB	The TVB count increased	[130]
Nisin	Garlic extract	Milk	*L. monocytogenes* ATCC 7644; *S. Enteritidis* SE86; *E. coli* ATCC 8739; *S. aureus* ATCC 1901	Inhibition of bacterial growth	[131]
Nisin	Thymoquinone	Sterilized milk	*L. monocytogenes* ATCC 19115 and ATCC 15313	Damage membrane integrity, inhibit bacterial growth	[132]
Nisin	Green tea extract	Beet leaves	*E. coli* ATCC 25922	Inhibit the growth of bacteria and improve the texture and sensory properties of food	[133]
Nisin	*Laperrinei* leave extract	Camel meat	—	Inhibit the rapid increase of thiobarbituric acid value and ferromyoglobin	[134]
Nisin	Curcumin	Rainbow trout fillet	LAB	Retards bacterial growth	[48]

### 3.3. Bacteriocins and Organic Acids (OAs)

OA refers to acidic organic compounds containing carboxyl groups (excluding amino acids) and which are soluble in water or ethanol; they exist in a wide range of organisms [136,137]. The antibacterial mechanisms of OAs mainly include: (i) destroying the structure of the bacterial cell wall and cell membrane; (ii) reducing the pH of the intracellular environment; and (iii) causing extracellular acidification [138,139]. It has been found that OAs can inhibit common pathogens in farm-fresh products [140]. Six strains of *E. coli* inoculated on pea sprouts were exposed to 0.2 mol/L of ascorbic acid, citric acid, and malic acid for 10 min, resulting in cell membrane damage, reduced cell proliferation, and imbalance in amino acid anabolism and catabolism [141]. Other researchers found that 1.5% acetic acid, 1% citric acid, and 1.5% lactic acid interfered with energy metabolism, amino acid metabolism, and carbohydrate metabolism, including the TCA cycle, glycolysis, and biosynthesis of amino acids of *Salmonella enterica* strains (ATCC 6962, ATCC 13076, and ATCC 14028) inoculated on cucumber slices [142]. The effectiveness of bacteriocins as biopreservatives is often compromised due to environmental stresses such as harsh temperature and pH [143]. It has been found that bacteriocins combined with OAs have enhanced antibacterial effects. The synergies of bacteriocins and OAs in food substrates are summarized in Table 4.

For a long time, food spoilage caused by *B. subtilis* has attracted the attention of researchers in the food industry [144]. The combination of nisin and OA has been proved to have potential applications in potatoes and meat to prevent and control spoilage caused by *B. subtilis*. The mixture of acetic acid, propionic acid, and nisin can be used as a preservative against *B. subtilis* in meat and potatoes within 10 days of storage [145]. Studies have shown that nisin combined with formic acid effectively inhibits the proliferation of *B. subtilis* on potatoes and prevents severe discoloration due to bio-deterioration caused by the action of the spoilage bacteria [144]. In the past decades, combinations of bacteriocins and OAs have been proven to have strong antimicrobial activity and to protect various food from food-borne pathogenic bacteria and spoilage bacteria [146]. Co-treatment of 22.5 μg/mL nisin with 250 μg/mL free fatty acid of lauric acid or N-tridecanoic acid was able to improve the killing effect of biofilm cells. Compared with nisin or fatty acids alone, co-treatment with nisin and fatty acids resulted in a significant decrease in the number of biofilms and cells of *L. monocytogenes* (ATCC15313 serotype 1/2a and ATCC19115 serotype 4b) [147]. Zhao et al. found that a combination of nisin and citric acid caused *S. aureus* ATCC 29213 and *L. monocytogenes* ATCC 19115 cells to show a distorted and irregular shape. In addition, the same combination led to the release of cell contents (K^+^, ${\mathrm{PO}}_{4}^{3-}$, DNA, RNA) of *S. aureus* and *L. monocytogenes* [148]. Treatment with nisin and phytic acid (PA) in combination showed favorable synergistic bactericidal activity against *E. coli* O157:H7 by dissolving cell biofilms and destroying membrane integrity. In cold-stored beef refrigerated at 4 °C for 5 days, it was found that the bactericidal effect of the nisin–PA combination on *E. coli* O157:H7 was better than that of nisin or PA alone [149].

These findings indicate that combinations of bacteriocins and OAs can be used as new promising food biopreservatives while overcoming the resistances of foodborne pathogenic and spoilage bacteria. A co-treatment strategy with nisin and OA can destroy the bacterial cell membrane, making it easier for bacteriocins and organic acids to enter cells and bind to DNA, thereby acting as synergistic inhibitors [150]. This combination can be used as an alternative preservative in the food industry for the preservation of fruits and vegetables, meat products, and other food preservation substrates in the food industry.

**Table 4:** Application of bacteriocins in combined action with organic acids (OAs).

Bacteriocins	OA	Food System	Target Microorganisms	Result	References
Nisin	Acetic and propionic acids	Meat and potato	*B. subtilis* ATCC 6633; *S. aureus* ATCC 6538; *E. coli* ATCC 8239	Inhibit bacterial growth, delay food spoilage	[145]
Nisin	Formic acid	Potato	*B. subtilis*	Inhibit the proliferation of bacteria and color change	[144]
Nisin	Free fatty acid	—	*L. monocytogenes* (ATCC15313 and ATCC19115)	Destroyed the cells in the *L. monocytogenes* biofilm	[147]
Nisin	Lactic acid	Enoki mushrooms	*L. monocytogenes* (IL-1, ScottA, and IL-1); *E. coli* O157:H7(ATCC 43889, ATCC 43894, and ATCC 13895)	Inhibition of bacterial proliferation	[146]
Nisin	Citric acid	Pasteurized milk	*S. aureus* (ATCC 29213); *L. monocytogenes* (ATCC 19115)	Cell surface damage, cell contents (K^+^, ${\;\mathrm{PO}}_{4}^{3-}$, DNA, RNA) release, inhibit bacterial proliferation	[148]
Nisin	Phytic acid	Cold-stored beef	*E. coli* O157:H7	The cell biofilm is dissolved	[149]

### 3.4. Bacteriocins and Other Substances

In addition, bacteriocins can be combined with sucrose laurate, ethylene diamine tetra-acetic acid (EDTA), ε-polylysine, and other substances to enhance their antibacterial effects (Table 5). Under the combined action of nisin and sucrose laurate on *S. aureus* ATCC 25923, the protoplast of *S. aureus* was found to leak, the cell membrane ruptured, and intracellular substances leaked out [151]. Nisin and gilaburu (*Viburnum opulus* L.) caused DNA and protein leakage of *S. aureus* NCTC 10788 at pH 5 [152]. It was found that EDTA could effectively control food spoilage caused by oxidation. When combined with EDTA, nisin exhibited broad-spectrum antibacterial activity against most gram-positive and gram-negative foodborne pathogens [153]. Leelaphiwat et al. used poly (butylene adipate terephthalate) and thermoplastic starch blends as the basis to prepare an active packaging film loading nisin and EDTA through a blow film extrusion process. Their results showed that the membrane loaded with EDTA and nisin effectively inhibited fat degradation in pork tissue, and that the amount of TVC and LAB was lower than with a membrane loaded with nisin alone [154]. In addition, EDTA has been approved by the FDA as a food additive to prevent the oxidation of meat products [155]. Bacteriocin-producing *Lactobacillus curvatus* CRL705 and *L. lactis* CRL1109 in combination with Na_2_EDTA were able to inhibit the growth of *E. coli* O157:H7 in frozen ground beef patties [156]. The addition of nisin and EDTA to the chitosan–polylactic acid matrix remarkably improved the antibacterial activity of the fabricated chitosan–polylactate plastic film against *S. aureus* BCRC 10,451 and *E. coli* BCRC 11,634, and did not affect the mechanical properties of the film [157].

In addition, combinations of bacteriocins and certain polypeptides could enhance their bacteriostasis, and could be used as biological preservatives in the food industry. For instance, nisin combined with γ-aminobutyric acid has been shown to prolong the storage time of pork and strawberries [158]. In co-treatment with nisin and ε-polylysine (ε-PL), nisin destroyed the cell wall and cell membrane of bacteria, allowing ε-PL to enter cells and interact with their DNA, preventing DNA replication and inhibiting ATPase activity in a synergistic antibacterial effect on *S. aureus* ACCC 10141, *E. coli* ATCC 25923, and *B. subtilis* ACCC 10242 [159]. At present, plant-based meat products have attracted wide attention in food production and processing [160]. However, plant-based meat products are easily contaminated by bacteria during storage [161]. Therefore, researchers are paying close attention to biological preservatives. For example, a blends of 1% chitosan, 2.5% tea polyphenols, and 0.04% nisin was able to delay the increment of *S. aureus* and *E. coli* in plant-based meat made from soybean protein isolate, pea protein, and wheat gluten [162]. Previous studies have confirmed that nisin combined with sesamol can synergistically destroy the cell membrane of *L. monocytogenes* ATCC 19112. As a result, the surface zeta potential and conductivity of the cell membrane of *L. monocytogenes* ATCC 19112 after combined treatment for 6 h were significantly higher than after treatment with nisin or sesamol alone for 6 h. Moreover, the combination of nisin and sesamol was able to completely inhibit the growth of *L. monocytogenes* ATCC 19112 in pasteurized milk within 48 h [52].

**Table 5:** Application of bacteriocins in combined action with other substances.

Bacteriocins	Other Substances	Food System	Target Microorganisms	Result	References
Nisin	Sucrose laurate	Milk beverage	*S. aureus* ATCC 25923	Cell wall damage, cell morphology damage	[151]
Nisin	*Viburnum opulus* L.	—	*S. aureus* NCTC 10788	DNA, protein leakage	[152]
Nisin	EDTA	Pork	TVB; LAB	Retarded bacterial growth and inhibited lipid degradation	[154]
Bacteriocins form *L. curvatus* CRL705 and *L. lactis* CRL1109	Na_2_EDTA	Frozen ground-beef patties	*E. coli* O157:H7	Decrease in bacterial population	[156]
Nisin	EDTA	Fish fillet	*E. coli* BCRC 11,634; *S. aureus* BCRC 10,451	The antibacterial activity of the membrane was improved	[157]
Nisin	γ-Aminobutyric acid	Pork and strawberry	—	Prolong storage time	[158]
Nisin	ε-Polylysine	Frankfurter-type sausage	TVC; *B. subtilis*	Most of the outermost layer of bacterial cells disappeared	[159]
Nisin	Chitosan, Tea Polyphenols	Plant-Based Meat	*E. coli*; *S. aureus*	Inhibition of bacterial growth	[162]
Nisin	Sesamol	Pasteurized milk	*L. monocytogenes* ATCC 19112	Surface zeta potential and conductivity rise	[52]

## 4. Conclusions and Perspectives

Food safety is a vital issue that affects public health around the world. Eating food contaminated with foodborne pathogens and spoilage bacteria such as *S. aureus*, *L. monocytogenes*, or *E. coli* can cause serious illness. A number of physical and chemical preservative methods, such as reducing water activity, adjusting pH value, and adding preservatives, are used to reduce microbial risks and extend the shelf life of food. As people are paying more and more attention to personal health, the drawbacks of chemical preservatives and processing technology are being gradually exposed.

For the sake of food safety and quality, bacteriocins have attracted great attention as natural alternative preservatives. However, bacteriocins interact with food components such as proteins and lipids, which can affect their antibacterial efficiency. It is necessary to increase the amount of bacteriocin to effectively inhibit the growth of pathogenic bacteria; however, excessive amounts may affect the sensory quality of food. Bacteriocins can be combined with chemical substances as an effective strategy to hinder the growth of corrupt microorganisms. Studies have shown that bacteriocins in combination with other chemical substances can inhibit the proliferation of foodborne pathogens and spoilage bacteria by destroying the cell membrane structure, inhibiting amino acid transport, and disrupting the proton motive force.

A great deal of work has been carried out to analyze the properties of various bacteriocin combinations; however, there is much work yet to be done in order to comprehensively and systematically reveal the mechanisms of action of these combinations of bacteriocins and chemical substances. In addition to the development of advanced techniques to facilitate the purification and characterization of new bacteriocins, it is necessary to carry out systematic in vivo research in order to evaluate the biological activity and safety of jointly acting bacteriocins and chemical substances in practical applications, including their absorption, acute and subacute toxicity, immunogenicity, and side effects. At the same time, the pathway of action of bacteriocins in combination with multiple chemical substances need to be further studied, as do the mechanisms of bacteriocins in combination with chemical substances and physical treatment methods and the range of bacteria targeted by bacteriocins in the complex microbiome environment. In addition, in order to reduce production costs and improve the effectiveness of bacteriocins, the combination of bacteriocins and chemical substances with different concentrations can be customized according to different food substrates to improve the bioavailability of bacteriocin while ensuring that the sensory qualities and attributes of the food itself are not changed. Exploring the synergistic or antagonistic relationships between different kinds of bacteriocins and chemical substances can help to obtain more effective antibacterial substances in order to exert better antibacterial activity. Studies have found that bacteriocins combined with certain substances are not suitable for food substrates. Future research could further explore applications to other industries such as biology and medicine. It is important to actively explore the combination of bacteriocins and other antimicrobials in order to combat currently undertargeted pathogens. Encapsulation processes such as liposome entrapment, coacervation, electrospinning, emulsification, vibrating technology, and spray-drying have been adopted to protect active substances and improve the stability of bacteriocins and chemical substances during use. Bacteriocins are essential for regulating gut health in humans and animals. However, it is a challenge to integrate bacteriocin-producing probiotics into food and feed and to ensure their antibacterial activity during processing and subsequent passage through the gastrointestinal tract of the host. In addition, the antibacterial effects of bacteriocins on foodborne pathogens are related to the dosage, and a high concentration of bacteriocins may have negative effects. In future research, it is necessary to improve the stability and antibacterial activity of bacteriocins while actively exploring new bacteriocins with high bacteriostatic effects on gram-negative bacteria. Overall, additional research is needed to strengthen knowledge on bacteriocins in the fields of genetic engineering and biotechnology.

## Figures and Tables

**Table 1:** Classification of bacteriocins.

Classify	Characteristic	Source	Represent	References
Class I	A modified peptide containing 19–50 amino acids.	*L. lactis* *Bacillus*	Nisin Subtilin L-Q 11	[80,81]
Class II	Unmodified membrane active peptide with molecular weight less than 10 kDa.	*Pediococcus acidilactici*	Pediocin PA-1 Pediocin L50	[82,83]
Class III	Thermal instability, high molecular weight > 30 kDa	*Lactobacillus*	Helveticin J	[84]
Class IV	A complex protein composed of one or more chemical groups.	*Lactobacillus*	Lactocin 27	[85]

## Data Availability

No data was used for the research described in the article.
